# Medical Extracts

**Published:** 1885-03

**Authors:** 


### Medical Extracts.

### On the Use of Cocaine in Chronic Pharyngitis.

Dr. Jahn, of Grevesmühlen, reports, in the *Deutsche Medizinal-Zeitung* of March 9th, 1885, that he has found the following mixture, containing cocaine, of great value in the treatment of certain forms of primary chronic pharyngitis:—

| | | |
|---|---|---|
| Cocaine ... ... ... ... ... | ·10 | gramme. |
| Glycerine ... ... ... ... ... | 15·00 | " |
| Acid Carbolic ... ... ... ... | ·01 | " |
| Aq. dest. ... ... ... ... ... | 35·00 | " |

He painted this solution on the pharynx once or twice a day, and found that when it was done in the evening, the vomiting which so frequently ensues as the result of the effort to cough up the small plugs of mucus characteristic of the disease did not take place on the next morning, and the irritability of the throat was less. The effect appears to have lasted about eight or ten hours, when the painting should be repeated.

Dr. Jahn also suggests its use in the vomiting of phthisis, where he thinks it will supersede Woillers's use of strong solutions of bromide of potash.

The carbolic acid in the mixture is added simply to enable it to be kept undeteriorated for a considerable time.

### Cocaine in Affections of the Nose, the Throat, and the Ear.

Dr. S. von Stein, writing in the *Med. Obosrenige*, says that he has used a $5^{\circ}/_{0}$ ointment, partly made with pure cocaine and partly with the hydrochlorate. He prefers to use the drug in the form of ointment rather than in that of a solution, as the former remains longer in contact with the mucous membrane, and so is most economical; and also the bitter taste of the drug is not so noticeable, which is an advantage in dealing with children and sensitive patients.

Dr. S. has used it successfully in laryngitis (acute, chronic, and phthisical), the swelling and pain being lessened by it; rhinitis (acute and chronic); and in acute otitis media, with perforations of the membrane.

Also, he has used it to anæsthetise the inferior part of the nasal forsa, and so rendered it easy to catheterise the Eustachian tube in sensitive subjects. He found that it was of especial value in the diagnosis of an exudation in the middle ear. After dropping into the outer ear a solution of 1 to 5 $_{0}/^{0}$, the tympanic membrane becomes pale, and the promontory can be seen through it in the healthy ear. The membrane is not, however, anæsthetised, but only somewhat less sensitive. In a case of perforation of the membrane, Dr. S. observed that after dropping some of the above solution, the mucous membrane of the middle ear also became pale. No ill consequences ever ensued.—*Deutsche Med.-Zeit.*, March 12th, 1885.

### Periodically Recurring Paralysis of the Oculo-motor Nerve.

Dr. P. J. Mobius, of Leipsic, reports the case of a girl, six years of age, in whom total paralysis of the right oculo-motor nerve took place every year since she was eleven months old. It was accompanied by vomiting and severe pain in the eyes of some days' duration, and gradually disappeared in eight or ten weeks, with mydriasis. The attacks have been more severe as she has grown older. The first lasted three days; some of the following, eight or nine weeks; the last one, ten weeks. Between the attacks the child is lively; but the mother states that she is timid, and at times jumps up in the night as if she were insane, and once tried to jump out of the window. The attacks of paralysis appear to come on very gradually, as was also noticed by Von Hasner and Saundby in their cases. The periodicity, as in some other nervous affections, migraine, &c., is not explainable. As to the seat of the affection, Dr. M. places it in the region of the root of the oculo-motor nerve, and especially because of the accompanying headache and vomiting, symptoms which are not found in peripheral paralysis on the one hand, and are observed in cerebral paralysis on the other. The pain is produced by irritation of the "descending root of the trigeminus," which is situated near the nucleus of the oculo-motor, and the former must (seeing that the pain is distributed over the forehead, and even the whole of one-half of the head) contain sensory fibres for the dura mater, as well as for the eye itself. In this way the pain of migraine, or of cerebral tumour, can be better explained by supposing an irritation of the "descending roots" rather than of the dura mater itself. The writer ascribes the periodically recurring oculo-motor paralysis to an anatomical permanent, possibly slowly progressing, lesion in this region of the brain. The fact that the pain ceases when the paralysis begins may be thus explained: at first a swelling of the affected part exists and compresses the tissue in its neighbourhood; then later on this ceases; therefore, if the swelling be absent, then the pain is absent. We may, the author considers, thus explain why chronic, especially simple atrophic, processes may go on in the region of the root of the oculom-otor nerve without pain.—*Deutsche Med.-Zeit.*, March 5th, 1885.

### Notes on Two of the Newer Remedies.

Dr. Leubuscher, of Jena, reports the following, from Professor Rossbach's *Clinic:*

1. Diathylacetal (Acetal).—This drug has been tried, especially in asylums, and most observers are agreed that it is not of much value in inducing sleep or quieting the patients; in fact, it was found that it made them more restless. Given in chorea, a child of ten years old took 5 grammes without effect, then it took 7·5 grammes, and in about one minute afterwards redness of the skin appeared, at first noticeable here and there on the face, but gradually becoming more and more diffusely spread over shoulders, chest, and extremities, in the latter especially in the region of the joints. The spots were not raised above the level of the surrounding skin, nor were they painful to pressure; but they felt hotter than the unaffected parts. Salivation ensued. Pupils moderately dilated; reacted to light well; heart much excited; pulse 144. The redness of the skin disappeared after four or five hours.

2. Oleum Gaultheriæ.—In a large number of cases of acute rheumatism which were treated with the above, a prompt effect was produced in every case, just as would be produced in similar cases by salicylate of soda; noises in the ears and deafness were also noticed on using ol. gaulth., just as in sod. salicyl. Within a short time the appetite was entirely destroyed, and the digestive system injuriously affected. The dose was 16 minims in capsule every two hours.—*Deutsche Med.-Zeit.*, Dec. 25th, 1884.

[Dr. B. J. Baron would like to add that he has tried the above drug at the Bristol General Hospital, in several cases of acute rheumatism, and has found that it is a very promptly-acting anti-pyretic in this disease, the temperature coming down to normal in a very short time, and not rising again, except under exceptional and otherwise explainable circumstances; the pain in the joints also is rapidly lessened. As to the effect on the heart, the usual cardiac complications did not appear to be affected differently from what they are under the use of salicylate of soda. Vomiting was not produced by ol. gaulth., as it so often is by sodæ salicyl.; but most of the patients complained very much of the disagreeable taste of the drug. Noises in the ears and deafness were not produced so frequently as when the salicylate was used; still, these disagreeable effects were observed very typically in one or two cases. The drug was given in 10-drop doses in an emulsion, every hour for four or five hours, and then every two hours; the intervals being lengthened after 24 to 36 hours, the dose remaining the same.]

### Carnrick's Beef Peptonoids.

The value of this preparation has been well shown by Dr. A. Stutzer, of Bonn, in his report on the various preparations of meat now in the market.

It is a fine dry powder, made up of meat, wheat gluten, and vaporised milk; it is accordingly a mixture of easily digested nitrogenous materials of high nutritive value. Chemical analysis shows that one hundred parts by weight contain as much peptone and readily digestible albumen as in 898 parts of Liebig's extract of beef. It also contains 10.67 per cent. of fatty matter, and 10.02 per cent. of soluble non-nitrogenous carbo-hydrates.

Its chemical composition should give this combination a very high position in the dietary necessities of the sick-room; it requires only to be tested, and its merits are at once realised, inasmuch as patients take it readily, find no fault with it, and the results show that it has a real nutritive value.

### Malignant Pustule cured by intravenous injections of Phenic (Carbolic) Acid.

J. P., æt. 29, of robust constitution, came under my notice on the 19th of April last, suffering from six malignant pustules, three of them on the right forearm, one on the right hand, one on the left temple, and one on the neck, this last giving rise to a considerable amount of swelling. The patient was in a high fever, suffering from dyspepsia and a general feeling of illness. He stated that three weeks previously he had eaten of the flesh of an ox that was found dead in a field. On the 14th of April, sixteen days after eating the flesh, he noticed that the glands of the neck began to swell. On the 17th he was in a high fever, and the swelling extended to the chest and face; he then noticed the six pustules.

After consulting with Dr. Yturrizaga I incised the pustules and applied nitric acid, giving the patient a tonic and anti-spasmodic mixture. On the following day the patient was much worse, the swelling had increased, causing dyspnœa and dysphagia; he was very restless. I decided to inject a solution of phenic acid into his veins. I took one grain of phenic acid and dissolved it in 200 minims of water; of this solution I injected 50 minims into three veins. After a few hours I noticed a great improvement; I gave him two more injections. On the following day the improvement continued; gave him two more injections. On the third day the fever had left him and the swelling was considerably reduced. From this time the patient steadily recovered. In a few days he was quite well, there only remaining a small superficial abscess on his neck.

I learn that five others who had eaten of the same flesh developed pustules and all died.—Dr. TOMAS SALAZAR, *La Cronica Medica*, Lima, Peru.

### Locomotor Ataxia and Syphilis.

M. Fournier, in his recent volume presented to the French Academy of Medicine, entitled *Leçons sur la période præataxigue du tabes d'origine syphilitique*, annexes an analysis of 249 cases of locomotor ataxia, in 231 of which he was able to elucidate a history of syphilis as an antecedent. It follows that in 100 cases of ataxia syphilis was found to be an antecedent etiological factor in 93.—*Bulletin de L'Académie de Médecine*, No. 53, 1884.

### The Liberating of the Ring Finger, in Musicians.

When the middle finger and the ring finger are brought down by the flexor muscles, and their balls are held down firmly against the keys of a musical instrument, as in performing on a piano, for the purpose of producing continuous sounds, and at the same time it should be necessary to extend and then to flex the ring finger in order to produce accompanying sounds, it will be found that in the still flexed position of the middle and little fingers, the ring finger can be but very slightly extended. Its complete extension, without operative interference, can only be brought about by long continued exertion in practice, when elongation of certain accessory, but restricting, tendons is made by nutritive change.

In the dorsal aspect of the metacarpal zone in man, dissection shows that the tendon of the extensor communis digitorum muscle that goes to the ring finger gives off a slip on either side, one of which goes to join the extensor tendon of the middle finger and the other to join the extensor tendon of the little finger. These two slips are known as the lateral vincula or *accessory* tendons. Now, while the middle and little fingers are held in a flexed position, these accessory tendons, by virtue of their attached extremities, hold in check the extending power of the muscular fibres operating upon the tendon of the ring finger, and thus this finger is restricted in its function of extension to a very limited degree.

Since 1857 I have divided these accessory tendons for the purpose of liberating the ring finger in fourteen persons, and in nine of these the operation was performed on the tendons of both hands at one sitting. I do not think at any one of these operations half a drachm of blood was lost. In not one of them did any accident follow the operation. The issue in all of them was successful.—Dr. W. S. FORBES, in *The Cincinnati Lancet and Clinic*, Dec. 27th, 1884.

### Management of New-born Infants.

The *Medical World* says: In the management of the new-born infant we are gradually approaching Nature's methods. In the maternity department of the Philadelphia Woman's Hospital the management of new-born babes has been as follows:

As soon as the head is born the eyes are washed with an antiseptic solution. When the body is born the child is left in the bed to await the expulsion of the placenta. No effort is made to remove the placenta under half or three-quarters of an hour; before this time it is generally expelled by Nature. When the placenta is expelled it is placed in a pan, and the child is wrapped up and laid away with the placenta still attached.

The child is now left and the attention is given to the mother. After the mother is cared for the child receives attention. By this time the pulsations in the cord have long since ceased. The cord is now cut and the blood "stripped" out of the stump, but neither end is ligated. The stump is not dressed, nor is any band put around the child's body. The child is neither washed nor dressed, only a diaper and a simple "slip" or gown is put on, and then it is warmly wrapped up and put in a little bed to itself. After twenty-four hours it is taken to the babies' bath-room (which is properly heated), and there it is washed and dressed.

Dr. Tyng, the physician in charge, tells us that since this plan has been adopted the babies get along much better. We were in the wards of this department about an hour, and during this time there was not a single cry from the babies. They all seemed contented and happy, and were doing well. We are convinced that washing the child immediately after birth, or keeping it half naked for a long time during the process of careful dressing, is not good practice.—*The Obstetric Gazette*, Cincinnati, Nov., 1884.

### The treatment of Chronic Ostitis of the Hip.

1. The "expectant" treatment is not, in an orthopædic or a surgical sense, any treatment at all. Cases that have no medical or surgical attendance whatever are followed, so far as my own observation goes, with just as good results.

2. Traction with motion is based upon a false pathology, and does not, in my opinion, do what its advocates claim for it. The motion is certainly not as great, as a rule, as one would be led to expect.

3. Fixation and rest, when properly carried out, yield better results, I believe, than any other plan.

4. The key-note in the treatment of ostitis of the hip is not the splint employed, not the crutch, or the high shoe, but it is the management of the case. Some men can get admirable results with any kind of splint. The case must be closely watched, the apparatus must be kept fully up to its duty, the indications must be met, and one must not grow impatient, because time is an important factor.

Let one be early impressed with the tediousness of the case, and let him also make up his mind that the case must be managed rather than treated with any special form of apparatus.—Dr. Gibney, *Philadelphia Medical Times*, Dec. 13th, 1884.

### Treatment of Diabetes.

We have received from a reader the request to give the latest and most approved treatment of diabetes, together with a diet list for this disease. We regret the limitation of the space and the time necessary to a full compliance with this request. We take this opportunity to state, however, that there are evidences that a very decided change is taking place touching the treatment of diabetic patients. It was the fashion, not so many years ago that even young practitioners are unable to recall it, to limit the treatment to the exclusion of such articles of food from the dietary as are converted, through physiological processes, into sugar. Experience has taught that it is not all of the treatment of diabetes to withhold saccharine and amylaceous food, and even that there are cases in which such withholding is directly detrimental. Such food is necessary to the building up of the tissues, whose exhaustion is one of the characteristics of the disease. Diabetes is a term which has come to be synonymous with glycosuria in the nosology of the general practitioner, and, doubtless, if the story were fully told, it would be found that this confusing of the two conditions has been the cause of much fatality. A patient who has sugar in his urine is not necessarily a sufferer from diabetes. But we cannot enter into a discussion of this phase of the question at this time. Diabetes proper is essentially a disease of nervous origin, and this fact cannot be overlooked in the successful treatment of it.

Among the drugs which have been found most beneficial are opium, codeia, ergot, and bromine, and cures are reported from their use by trustworthy authorities. Alkalies, bicarbonates, acetates, and citrates have also been recommended; and it is to the presence of these that the efficacy of the mineral waters of certain springs is, doubtless, traceable. In the treatment, however, there are no "specifics," and no one drug must be relied on to the neglect of the general constitutional conditions.

In such cases as seem to demand the "diabetic diet," so called, the following table by Pavy is, probably, as reliable as any:

DIETARY FOR THE DIABETIC.

*May Eat*—Butcher's meat of all kinds, except liver; ham, bacon, or other smoked, salted, dried, or cured meats; poultry, game; shell-fish and fish of all kinds, fresh, salted, or cured; animal soups not thickened, beef-tea, and broths; the almond, bran, or gluten substitute for ordinary bread; eggs dressed in any way; cheese, cream-cheese, butter cream; greens, spinach, turnip-tops, turnips,* French beans,* Brussels sprouts,* cauliflower,* brocoli,* cabbage,* asparagus,* seakale,* vegetable marrow,* mushrooms, water-cress, mustard and cress, cucumber, lettuce, endive, radishes, celery; vinegar, oil, pickles; jelly (flavoured, but not sweetened), savory jelly, blanc-mange made with cream, and not milk; custard made without sugar; nuts of any description, except chestnuts; olives.

NOTE.—Those marked with an asterisk (*) may only be eaten in moderate quantity, and should be boiled in a large quantity of water.

*Must avoid Eating*—Sugar in any form, wheaten bread and ordinary biscuits of all kinds; rice, arrowroot, sago, tapioca, macaroni, vermicelli, potatoes, carrots, parsnips, beetroot, peas, Spanish onions; pastry and puddings of all kinds; fruit of all kinds, fresh and preserved.

*May Drink*—Tea, coffee, cocoa from nibs, dry sherry, claret, dry Sauterne, Burgundy, chablis, hock, brandy, and spirits that have not been sweetened; soda-water; Burton bitter ale, in moderate quantity.

*Must avoid Drinking*—Milk, except sparingly; sweet ales, mild and old; porter and stout; cider, all sweet wines, sparkling wines, port wine (unless sparingly), liqueurs.—*The Therapeutic Gazette*, Dec., 1884.

### Explosive Drugs.

Several instances are related in the *Deutsche Medicinal-Zeitung* of 29th September, 1884, of injuries resulting from the explosion of compounds ordered in physicians' prescriptions. A gargle was ordered of chlorate of potassium, chloride of iron, and glycerine. It was prepared, and five minutes later the bottle exploded in the purchaser's pocket, wounding him quite severely with the fragments of glass. A mixture of hypophosphite of lime, chlorate of potassium, and lactate of iron, exploded and nearly killed the prescription clerk who was compounding it. Even the simple trituration of calcium hypophosphite is dangerous; a young pharmaceutist was killed by an explosion which was caused by the shaking of a solution of this substance. Physicians not unfrequently order a solution of chromic acid in glycerine. But when the acid is added quickly and all at once to the glycerine, a readily explosive substance like nitro-glycerine is formed. Chlorate of potassium when mixed with tannin or muriate of morphia often explodes. The combination of iodide and preparations of ammonia must be made cautiously, as iodide of nitrogen is formed, which explodes on the slightest touch. Indeed, one ought to be very careful in ordering and compounding mixtures in which easily reducible substances enter—such as the chlorates, the hypophosphites, the nitrates, preparations of iodine or ammonia, chromic acid, glycerine, permanganate of potash, &c. If physicians would but remember the danger of explosions in preparing such compounds, they would less often put the lives of druggists and of their own patients in jeopardy.—*Philadelphia Medical Record*, 29th Nov., 1884.

### Corn Cures.

M. P. Vigier has examined a number of the secret remedies recommended as cures for corns, and thinks that the following formula very closely resembles them:

℞ Salicylic acid, gr. xv.
Alcoholic extr. cannabis indica, gr. vijss.
Alcohol (90 per cent.), M xvi.
Ether, M xl.
Elastic collodion, gr. lxxv.

M. To be preserved in a tightly-corked flask.

In employing this remedy a brush or the end of a match stick is to be dipped into the mixture, and rubbed several times over the excrescence every other day for a week. After a few applications the corn is readily removed with the finger after a warm foot-bath.—*La France Médicale*, Nov. 22nd, 1884.

### The Physiological Action and Therapeutic Application of Boracic Acid.

Boracic acid is one of the valuable agents of the antiseptic method, particularly from its freedom from all irritant properties. It may, therefore, be employed in the antiseptic treatment of the transparent media of the eye, cases in which the use of carbolic acid would be entirely inadmissible. Its value in certain diseases of the ear, and as an injection (2 parts to 60 of water) in vesicle troubles accompanied by decomposition of the urine, is well known.

Although both the acid and its salt, borax, are enormously used in the arts and, to a considerable extent, in practical medicine, yet we have very little definite knowledge as to their physiological action. To fill up this hiatus Dr. F. E. Stewart recently performed in the laboratory of the University of Pennsylvania, under the supervision of Dr. H. C. Wood, a number of experiments, whose results are worthy of being put on record. An experimental difficulty is the insolubility in water of the acid and its ordinary salt. This was in part obviated by the use of the quadroborate of sodium, which was found to be much less soluble in water than is stated in Gmelin's *Chemistry*. Enormous doses of the drug were required to produce death in the frog. The notable symptoms were simply a progressive loss of muscular power and of reflex activity. So long as voluntary movement remained the poisoned batrachian showed evidences of sensation when disturbed, and there was not at any time any apparent anæsthetic influence.

The respiration ceased before the heart's action, and the drug may therefore be considered as a respiratory poison; but that it is not entirely without influence upon the frog's heart was shown by the fact that in some experiments a saturated solution of the quadroborate applied to the exposed heart arrested its action in five minutes, although in other trials a much longer time was required.

Our knowledge as to the action of this remedy has also been recently extended by M. C. Baumfeld in his inaugural thesis at Paris (*Thèse de Paris*, 1884), in which he demonstrated its great antiseptic value and energy as a disinfectant and parasiticide, and as a dressing for wounds of mucous surfaces. Eczema and impetigo in children, and intertrigo are rapidly relieved by boracic acid, either in powder mixed with starch or as an ointment.

As a powder mixed with starch it also serves to destroy the odour from the axillæ or feet, though here, as in all cases in which boracic acid is applied to the skin, it should be certain that it is perfectly free from mixture with other acids, particularly sulphuric or hydrochloric, otherwise irritant instead of calming effects will be produced.

The following formulæ are recommended for an ointment of boracic acid:

℞ Pure Boracic, 1 part.
White wax, 1 part.
Paraffine, 2 parts.
Almond oil, 2 parts.

Melt the wax and paraffine by heat together with the oil, and mix thoroughly in a warm vessel, with the boracic acid. (Lucas Champlonniére.)

℞ Oil of sweet almonds, 210 parts.
Paraffine, 60 parts.
White wax, 30 parts.
Boracic acid, 60 parts.

An ointment can be made in this manner which can be readily applied on muslin. (Lejeune.)—*Therapeutic Gazette*, January 15th, 1885.

### A New Symptom of Lead-poisoning.

M. Du Moulin has recently presented to the Brussels Academy of Medicine *(Rev. de Therep.)* a young man who five days previously was attacked with lead colic, but who no longer presented any apparent sign of lead poisoning, other than the blue line on the gums. He called attention to a very curious and new pathognomonic symptom which frequently appeared before the blue line of the gums, always accompanied it, and is more characteristic and better demonstrated than the other. This symptom manifests itself by the formation in the epidermis of a frequently very abundant deposit of sulphate of lead. By the application of an alkaline sulphate he had traced black lines all over the body of the subject presented. The reagent by the use of which he had inscribed the chemical symbol of lead (Pb.) on the chest, on the back and on the flanks of the subject, was a solution of monosulphuret of sodium, in the proportion 5 per cent., in distilled water. The sulphydrate of ammonia produced the same effect. He gave his experience as follows:

1. The skin of all persons affected with lead poisoning, so far as he had examined them, to the number of 14, contained lead in sufficient quantity to react directly upon the contact of a glass rod dipped in a solution of monosulphuret of sodium at 5 per cent.

2. In recent cases this reaction is much stronger than in older cases.

3. Washing with cold or hot water does no more than to remove a few epidermal scales containing lead; the limpid filtered liquid contains no lead in a soluble state.

4. Prolonged washing with tartrate of ammonia removes from the skin this property of blackening by the sulphuret of sodium. The water used contains all the lead in the form of a sulphate rendered soluble by the tartrate.

5. The sulphuret of ammonia and the monosulphuret of sodium precipitate a considerable quantity of lead, in the form of the sulphuret.

6. The surface washed by the tartrate of ammonia no longer reacts with the sulphuret of sodium; the deposit then which exists upon and in the epidermis is exclusively formed of sulphate of lead.

7. Those parts of the body, which from the prolonged washing with tartrate of ammonia no longer react with the sulphuret, resume this characteristic at the end of a few days.

8. The reaction, which is not very apparent at the end of one or two days, increases daily.

9. The sulphate of lead then passes to the skin and becomes fixed there through the agency of the cutaneous secretion; but we are still ignorant of how that body, so insoluble in its nature, is carried there and becomes so fixed.—*American Medical Association Journal*, Feb. 14th, 1885.

